# 3D bioprinting of conductive hydrogel for enhanced myogenic differentiation

**DOI:** 10.1093/rb/rbab035

**Published:** 2021-08-14

**Authors:** Ying Wang, Qingshuai Wang, Shengchang Luo, Zhoujiang Chen, Xiang Zheng, Ranjith Kumar Kankala, Aizheng Chen, Shibin Wang

**Affiliations:** 1 Institute of Biomaterials and Tissue Engineering, Huaqiao University, Xiamen 361021, P. R. China; 2 School of Pharmaceutical Engineering and Life Science, Changzhou University, Changzhou 213164, P. R. China; 3 Fujian Provincial Key Laboratory of Biochemical Technology (Huaqiao University), Xiamen 361021, P. R. China

**Keywords:** 3D bioprinting, conductive hydrogel, electrical stimulation, myoblasts, myogenic differentiation

## Abstract

Recently, hydrogels have gained enormous interest in three-dimensional (3D) bioprinting toward developing functional substitutes for tissue remolding. However, it is highly challenging to transmit electrical signals to cells due to the limited electrical conductivity of the bioprinted hydrogels. Herein, we demonstrate the 3D bioprinting-assisted fabrication of a conductive hydrogel scaffold based on poly-3,4-ethylene dioxythiophene (PEDOT) nanoparticles (NPs) deposited in gelatin methacryloyl (GelMA) for enhanced myogenic differentiation of mouse myoblasts (C2C12 cells). Initially, PEDOT NPs are dispersed in the hydrogel uniformly to enhance the conductive property of the hydrogel scaffold. Notably, the incorporated PEDOT NPs showed minimal influence on the printing ability of GelMA. Then, C2C12 cells are successfully encapsulated within GelMA/PEDOT conductive hydrogels using 3D extrusion bioprinting. Furthermore, the proliferation, migration and differentiation efficacies of C2C12 cells in the highly conductive GelMA/PEDOT composite scaffolds are demonstrated using various *in vitro* investigations of live/dead staining, F-actin staining, desmin and myogenin immunofluorescence staining. Finally, the effects of electrical signals on the stimulation of the scaffolds are investigated toward the myogenic differentiation of C2C12 cells and the formation of myotubes *in vitro*. Collectively, our findings demonstrate that the fabrication of the conductive hydrogels provides a feasible approach for the encapsulation of cells and the regeneration of the muscle tissue.

## Introduction

Tissue engineering mainly focuses on the fabrication of the substitutes for tissues and organs to overcome the challenges of insufficient donors or immune rejection, thereby restoring, maintaining and repairing the injured or diseased tissues [1–4]. Over the past decades, three-dimensional (3D) bioprinting has emerged as one of the most efficient microfabrication technologies in the tissue engineering field [5, 6]. In this context, hydrogels are often preferred as bioinks for the 3D culture of cells, owing to the high amounts of water, which not only offer excellent compatibility and reiterate the native extracellular matrices (ECM)-like environment but also highly suitable for printing toward the construction of macro-sized tissue-like constructs. These highly compatible hydrogel-based materials facilitate the interactions between cell matrix and maintenance of cell functions, such as proliferation, migration and differentiation of cells [7–9]. Among diverse biopolymeric hydrogels as bioinks, gelatin methacryloyl (GelMA), containing natural arginine-glycine-aspartic acid (RGD) sequence, can effectively mimic native ECM and promote cell attachment [10, 11]. In addition, the shaping and crosslinking process of GelMA hydrogel in terms of time and space can be successfully controlled by adjusting the irradiation of ultraviolet (UV) light [12]. Thus, GelMA can be considered as the ideal biological material for the 3D culture of diverse cell types [13, 14].

Indeed, biomaterials are designed to provide an external environment for the encapsulated cells *in vitro* [15–18]. For example, to engineer a functional muscle *in vitro*, the biomimetic materials create a conducive microenvironment for expanding mouse myoblasts (C2C12) and fusion of single-nucleated C2C12 cells [19, 20]. Moreover, the differentiation of C2C12 cells can result in multinucleated myotubes. Following the formation and maturation of myotubes, myofilament organization, as well as ECM remodeling, the muscle tissue enters the final phase of the regeneration process [21–23]. Despite the success in harboring the cells, the growth and function of cells are affected by the required electrical signals in muscle tissues [24, 25]. Basically, the bioelectrical character of the applied biomaterial may directly influence the migration and fusion of C2C12 cells. Despite the success in harboring the cells, the growth and function of cells are affected by the required electrical signals in some of the tissues, for instance, myoblasts [26]. To overcome these limitations, it is required to fabricate conductive biomaterials, which can be used to detect electrical signals and provide electrical stimulation (ES) subsequently for the encapsulated cells to regulate the biological activities. However, most biomedical hydrogels are nonconductive, leading to difficulties in transmitting electrical signals in the encapsulated cells [27]. Owing to these attributes, several reports have been focused on the fabrication of electroconductive hydrogels (EHs), combining the bionic properties of the hydrogels and the electrochemical characteristics of the conductive materials. On the one hand, the printed hydrogel-based scaffold could maintain rich hydrophilicity and sufficient mechanical properties within 3D porous architecture. On the other hand, the high electrical conductivity and electrochemical redox characteristics could be achieved due to the presence of conductive materials [28–30]. To illustrate these aspects, several kinds of conductive materials have been employed for the preparation of EHs, including noble metal nanoparticles (NPs) with high conductivity, carbon-based nanomaterials, such as graphene and fullerene [31–34]. Dispersing these conductive materials in biomedical hydrogels can enhance the conductivity of the hydrogel and increase the mechanical strength of the hydrogel scaffold [35]. However, high concentrations of noble metal-like and carbon-based materials sometimes lead to specific cytotoxicity issues. Accordingly, the selection of biocompatible conductive polymers should be carefully considered for biomedical applications.

Recently, the application of various conductive polymers, such as polyaniline (PANi), polypyrene, and polythiophene, has been widely investigated [24, 25, 36, 37]. The conductivity of these polymers comes from the conjugated double bonds along the main chain and π electrons in the main chain that can delocalize to form a conductive band [38, 39]. In addition, these polymers can be employed by incorporating them within the hydrogel matrices as 3D scaffold materials. For example, Soyoung *et al.* demonstrated the fabrication of electrically conductive hydrogel micropatterns based on PANi within poly(ethylene glycol) (PEG) hydrogel matrix (PANi/PEG) [25, 40, 41]. C2C12 cells were then cultured on the PANi/PEG substrate, exploring the effective differentiation of C2C12 cells and improved alignment of myotubes [25]. Among various conductive polymers, poly-3,4-ethylene dioxythiophene (PEDOT), as a type of polythiophene, displays a superior conductivity and electrochemical stability due to the presence of the ethylenedioxy groups at the 3, 4 positions of thiophene as well as excellent biocompatibility [40, 41]. For instance, Gong *et al.* designed a micro-grooved and highly conductive PEG/PEDOT hydrogel for the culture of C2C12 cells, presenting excellent cell proliferation and effective cell alignment [37]. Among various conductive polymers, PEDOT, as a type of polythiophene, has shown good biocompatibility [40, 41]. The ethylenedioxy groups at the 3, 4 positions of thiophene make PEDOT a superior conductive and electrochemically stable material. However, the water insoluble property of PEDOT limits the direct combination into a high-resolution complex scaffold structure, hampering the PEDOT application in tissue engineering [42].

**Figure 1.**
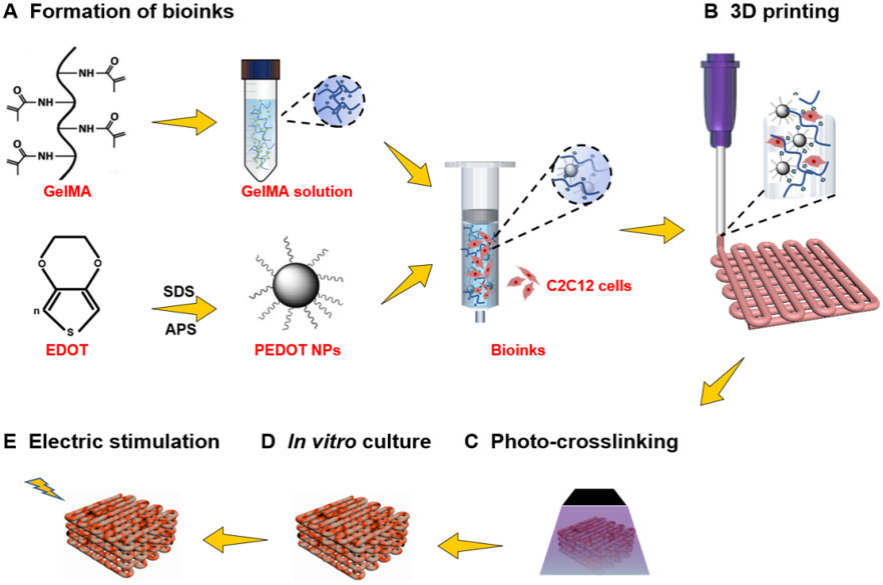
Outline of the method. Schematic illustrating the development of the conductive hydrogel based on GelMA for enhanced myogenic differentiation. (**A**) The PEDOT NPs were fabricated and subsequently dispersed into the GelMA hydrogel aqueous solution. (**B**) The hybrid bioinks compromised PEDOT NPs and C2C12 cells within the GelMA solution. (**C**) The 3D printing process of the GelMA/PEDOT conductive hydrogel scaffold with the encapsulation of C2C12 cells, as well as (**D**) the cell culture *in vitro* and (**E**) ES process.

Considering these issues, conductive scaffolds based on GelMA and PEDOT NPs were fabricated using the 3D bioprinting technology to encapsulate mouse myoblasts (C2C12 cells) and enhance the myogenic differentiation of C2C12 cells. As shown in Fig. 1, a conductive hydrogel by dispersing PEDOT NPs into GelMA solution was prepared. Further, the swelling ratio, conductivity and cytotoxicity properties of the fabricated hydrogels were investigated after UV irradiation. Then, the GelMA/PEDOT conductive scaffold encapsulated with C2C12 cells was printed by 3D bioprinting technology. The proliferation of C2C12 cells in the GelMA/PEDOT hydrogel scaffold and the effects of ES on the differentiation of C2C12 cells in the scaffold were further analyzed.

## Materials and methods

### Materials

Gelatin, 3,4-ethylene dioxythiophene (EDOT) and lithium phenyl-2,4,6-trimethylbenzoylphosphinate (LAP) were purchased from Aladdin Reagent Co., Ltd (Shanghai, China). Cell Counting Kit (CCK)-8 and Dulbecco’s modified Eagle medium (DMEM) culture medium were obtained from Keygen Biotech Corp., Ltd (Nanjing, China). Fluorescein isothiocyanate-phalloidin detection kit and Calcein-AM/propidium iodide (AM/PI) were obtained from the Solarbio Science and Technology Co., Ltd (Beijing, China). Sodium dodecyl sulfate (SDS) and ammonium persulfate (APS) were purchased from Maclean Biochemical Technology Co., Ltd (Shanghai, China).

### Synthesis of GelMA

GelMA was synthesized following the reported procedure [43]. Briefly, gelatin (10 g) was dissolved in carbonate buffer (100 ml, 0.25 mol/l) at 50°C, and the pH of the solution was adjusted to 9 using NaOH (5 mol/l) and HCl (6 mol/l). Then, the methacrylic anhydride (1 ml) was added and stirred for 3 h at 50°C. The pH of the solution was then adjusted to 7.4. Finally, the product was dialyzed, lyophilized and stored at −20°C until further use.

### Fabrication and characterizations of PEDOT NPs

Initially, EDOT (64 μl) and HCl (50 μl) were added to the SDS solution (20 ml, 0.01 mol/l) and stirred for 1 h at 40°C. Then, APS (600 μl, 2 mol/l) solution was slowly added to the above reaction mixture. Finally, the supernatant was removed after centrifugation at 12 500 rpm for 30 min and collect the samples [44, 45].

The morphology of PEDOT NPs was observed by TEM. FTIR and Raman spectroscopies (Renishaw, InVia, London, UK) were employed to analyze the functional groups of PEDOT during synthesis. In addition, the influence of PEDOT NPs addition on the chemical functionalities of GelMA hydrogel was determined using FTIR. The samples were scanned over a wavenumber region of 4000–500 cm^−1^. The UV-vis spectroscopic (Shimadzu, Kyoto, Japan) measurements were recorded in the wavelength range of 1100–300 nm.

### Fabrication and characterizations of GelMA/PEDOT composite hydrogel

GelMA/PEDOT composite hydrogel was prepared by the photo-crosslinking method. The prepolymer solutions were constituted of GelMA (6%, w/v), PEDOT NPs at different concentrations of 0, 0.1, 0.5, 1.0, 1.5, and 2.0 mg/ml, and LAP (0.5%, w/v). The prepolymer solution was exposed to UV light at 405 nm for crosslinking. The characterizations of the fabricated GelMA/PEDOT composite hydrogel were as follows.

Swelling ratio: The crosslinked GelMA/PEDOT composite hydrogel was freeze dried, and the mass of the dried sample was recorded as *W*_0_. Further, the freeze-dried sample was placed in ultrapure water for 24 h and re-recorded the weight again as *W*_1_. The swelling ratio was then calculated as *Q* = (*W*_1_ − *W*_0_)/*W*_0_.

Distribution of PEDOT NPs: SEM was applied to observe the porous architectures and distribution of PEDOT NPs in the GelMA/PEDOT composite hydrogel. The samples were freeze dried and sputter coated with gold before imaging.

Electrical conductivity: The electrochemical impedance of GelMA/PEDOT composite hydrogel was tested using an electrochemical workstation (CHI600E, Chenhua, Shanghai, China). The GelMA/PEDOT composite hydrogel was prepared in the shape of a thick disc (15 mm in diameter and 2 mm in thickness). The samples were placed between two copper sheets to form a sandwich structure with an alternating voltage of 5 mV. The scanning frequency was tested from 1–10^5^ Hz to get the electrochemical impedance spectrum.

Cytotoxicity: The CCK-8 kit was employed to investigate the cytotoxicity of the leach solution of GelMA/PEDOT composite hydrogel (2, 5, and 10 mg/ml) in C2C12 cells. In brief, the C2C12 cells were incubated with the leach solution for 24 and 48 h, along with the pure DMEM culture medium as the blank control. After incubation for predetermined intervals, the CCK-8 working solution (10 μl of CCK-8 in 100 μl of fresh medium) was added to the wells. After 2 h of incubation at 37°C, the relative growth rate (RGR) was analyzed following the equation:

RGR%=(sample group OD450 nm−blank group OD450 nm)/(positive control group OD450 nm−blank group OD450 nm)×100.

Rheological properties: Rheological properties of different solutions were analyzed using a rotational rheometer (Anton-Paar, MCR102, Graz, Austria). The viscosity values of GelMA hydrogels at various concentrations (4, 6, 8 and 10%, w/v) and GelMA/PEDOT NPs composite hydrogels at various PEDOT NPs concentrations (0.0, 0.5, 1.0, 1.5, and 2.0 mg/ml) were measured by changing the temperature from 17 to 40°C, respectively. Then, the storage (*G*′) and loss (*G*″) moduli of GelMA (6%, w/v) with the addition of PEDOT NPs at various concentrations were evaluated by changing the temperature from 5 to 40°C. Moreover, the shear stress–shear rate curve of GelMA (6%, w/v) compromising PEDOT NPs (1.0 mg/ml) was measured at 15 and 25°C, respectively.

Mechanical properties: The mechanical properties (complex storage modulus) of the crosslinked hydrogels were recorded using a rotational rheometer (Anton-Paar, MCR702, MultiDrive) at 25°C. Briefly, the samples were placed in individual wells of a 6-well plate (thickness of 2 mm) and immersed in the PBS under sterile conditions. For unconfined compression tests, vibration mode was chosen at 50 μm displacement and 1 Hz.

### 3D Bioprinting

The hybrid bioinks compromised homogenous mixed GelMA (6%, w/v), PEDOT (1.0 mg/ml), LAP (0.5%, w/v), and C2C12 cells (2 × 10^6^ cells/ml). The low-temperature cartridge with bioinks was installed on the 3D bioprinter (Bio-Architect, Jie Nuofei, Hangzhou, China), and the temperature of the printing platform was set as 4°C. Then, the scaffold encapsulated with C2C12 cells was bioprinted on the platform by adjusting the printing temperature (16–22°C) and extrusion pressure (0.06–0.12 MPa). After UV irradiation for 2 min, the sample was photo-crosslinked, transferred to a 6-well plate and cultured in a complete DMEM growth medium with fetal bovine serum (10%, w/v, Gibco, Grand Island) and penicillin–streptomycin (1%, v/v, Keygen) in the incubator (37°C, 5% CO_2_).

### ES procedure

The platinum electrode was fixed to the bottom of the petri dish with polydimethylsiloxane (Dow Corning, Michigan, USA), connecting to a pulse signal generator. The ES parameter was set using an electrical pulse with an amplitude of 5 V and a frequency of 1 Hz, and the stimulation was performed for 4 h per day [46].

### Proliferation of C2C12 cells in GelMA/PEDOT composite scaffold

The proliferation of C2C12 cells in the GelMA/PEDOT composite scaffold was analyzed by live/dead staining of the cells in the scaffold after 1, 3 and 5 days of incubation. Calcein-AM/PI (Keygen) staining kit was used for determining the cell apoptosis according to the manufacturer’s instructions. Additional assessments of the mean gray value of fluorescence were made by live/dead staining images at predetermined time intervals (1, 3 and 5 days) using Image J 1.8.0 (National Institutes of Health, Bethesda, USA).

### Differentiation of C2C12 cells in GelMA/PEDOT composite scaffold

To observe the morphology and distribution of C2C12 cells, F-actin and nuclei were stained by phalloidin and DAPI, respectively, within the bioprinted composite scaffold. Furthermore, desmin and Myogenin (Myog) immunofluorescence staining were performed to observe the protein expression of C2C12 cells encapsulated within GelMA/PEDOT composite scaffolds. The staining processes were carried out according to the manual instructions from the manufacturer. After staining, the GelMA/PEDOT composite scaffold with C2C12 cells was washed with PBS for 30 min and then observed under a confocal laser scanning microscope (CLSM, TCS SP5, Leica, Wetzlar, Germany).

## Results and discussion

### Fabrication and characterization of PEDOT NPs

**Figure 2.**
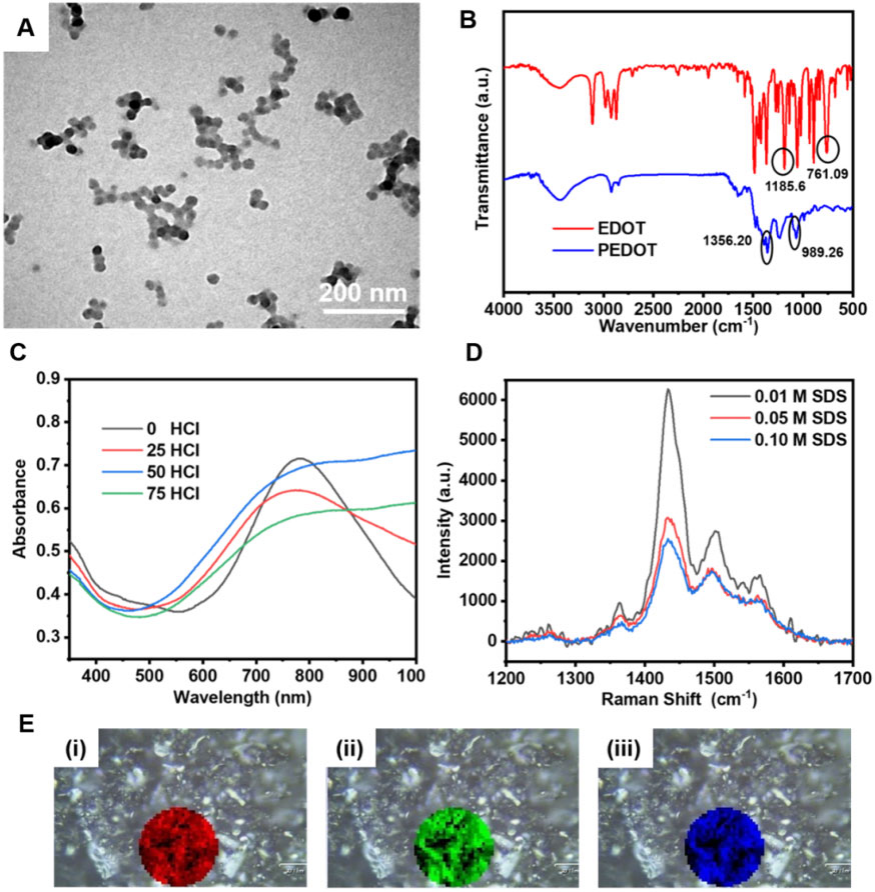
Characterization of PEDOT NPs. (**A**) Representative TEM image, (**B**) FTIR spectrum, (**C**) UV-vis spectrum, and (**D**) Raman spectra of PEDOT NPs. (**E**) Different ranges mapping scans of PEDOT NPs: (i) 1353–1375 cm^−1^, (ii) 1425–1443 cm^−1^ and (iii) 1493–1511 cm^−1^.

The particle size and morphology of the as-prepared PEDOT NPs were characterized by a transmission electron microscope (TEM, H-7100, Hitachi, Tokyo, Japan) (Fig. 2A). It was observed that PEDOT NPs were uniform, with an average diameter of around 30 nm. Further, the chemical functionalities of EDOT and PEDOT NPs were elucidated using Fourier transform infrared (FTIR, Nicolet iS50, ThermoFisher, Waltham, USA) spectroscopy (Fig. 2B). The spectrum of pure EDOT showed peaks at 1185 and 761 cm^−1^, which could be ascribed to the C–O–C bending vibrations of the ethylenedioxy and the Cα-H out-of-plane bending vibration, respectively [47]. In the spectrum of PEDOT NPs, the peak at 1356 cm^−1^ could be attributed to the C=C stretching vibration in the thiophene ring, and another peak at 1076 cm^−1^ ascribed to the C–S bond stretching vibration. Notably, the characteristic peak of Cα–H bond vibration in PEDOT disappeared, indicating that EDOT was polymerized by Cα-H. Moreover, the chemical structures of pure GelMA and GelMA/PEDOT NPs composite hydrogel were characterized (Supplementary Fig. S1). In the spectra of GelMA, the absorption peaks at 3309 and 1635 cm^−1^ could be attributed to C–N–C and C=C functional groups, respectively [48]. These two absorption peaks were also evident in the spectra of GelMA/PEDOT NPs composite hydrogel with a slight difference compared to that of the raw GelMA, indicating no significant changes of functional groups of GelMA with the addition of PEDOT NPs in the polymeric matrix. In addition, the UV-vis spectrum of PEDOT NPs revealed that the synthesized PEDOT NPs were all in the oxidized state (Fig. 2C). The absorption band around 600 nm could be due to the π–π electron transition. Moreover, the absorption band around 800–1000 nm could be correlated to the absorption of polaronic and bipolaronic states [49].

Furthermore, it was evident that adding a small amount of acid to the reaction system could increase the absorption of PEDOT around 800–1000 nm, indicating the enhancment in the degree of PEDOT oxidative polymerization. In the polymer chain, the Raman signals would be stronger with the increase of π–π bonds. The most substantial peak in the Raman spectrum of PEDOT NPs at around 1430 cm^−1^ could be due to the stretching vibration of the C=C bond corresponding to the PEDOT benzene structure. Moreover, the weaker peak near 1510 cm^−1^ could be attributed to the stretching vibration of the C=C bond corresponding to PEDOT quinone structure (Fig. 2D). The peak around 1360 cm^−1^ could be attributed to the stretching vibration of the C–C bond [49]. Evidently, the intensities of the Raman signals were increased with the decrease in the concentration of surfactant, indicating that the doping degree of PEDOT could be improved by increasing the concentration of surfactant. Consequently, the scanning range from 1353 to 1375 cm^−1^ could represent the distribution of C–C bonds, along with the scanning range from 1425 to 1443 cm^−1^, showing the distribution of C=C bond of PEDOT benzene structure. Moreover, the scanning range from 1493 to 1511 cm^−1^ could represent the distribution of the C=C linkage of the PEDOT quinone structure. Together, the functional group distribution of the synthesized PEDOTs was relatively uniform.

### Characterization of GelMA/PEDOT composite hydrogel

**Figure 3.**
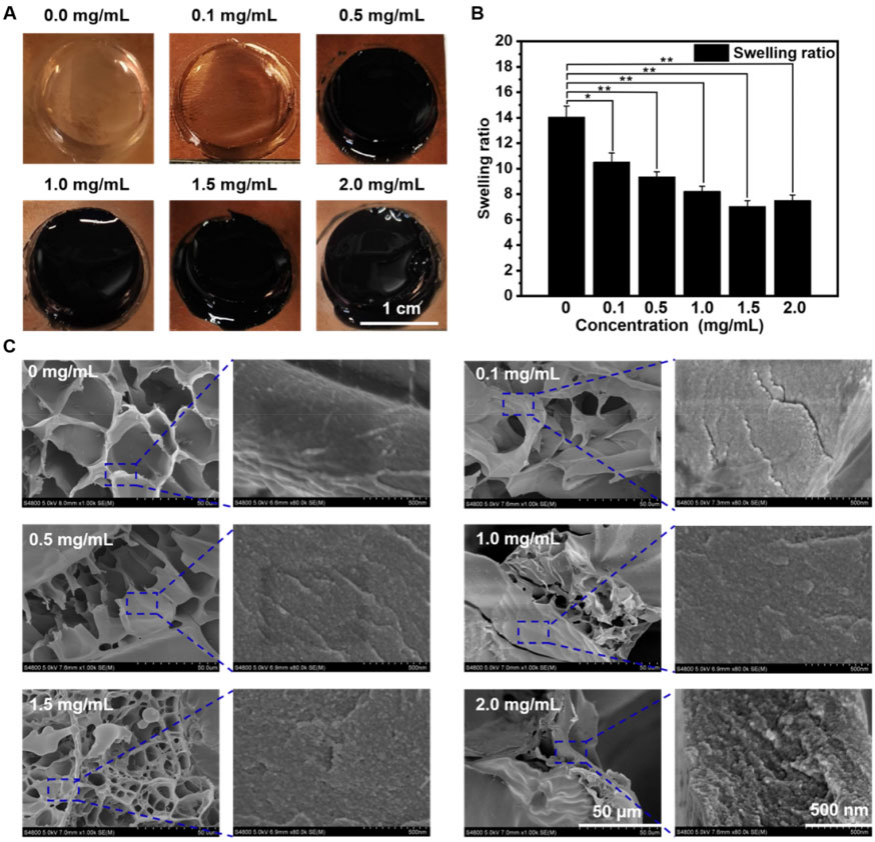
Characterization of GelMA/PEDOT hydrogels. (**A**) Photographs, (**B**) swelling ratio and (**C**) SEM images of GelMA/PEDOT hydrogels printed at different concentrations of PEDOT NPs (0.0, 0.1, 0.5, 1.0, 1.5, and 2.0 mg/ml).

GelMA/PEDOT composite hydrogels were filled into hydrogel discs with a diameter of 15 mm and a thickness of 2 mm. As shown in Fig. 3A, the GelMA hydrogel without PEDOT was transparent, while the color of the hydrogel gradually deepened and eventually turned to dark color with the increase of the content of PEDOT from 0.0 to 2.0 mg/ml. In addition, the color of each hydrogel disc was almost uniform at each concentration of PEDOT, indicating that the PEDOT NPs could be stable and uniformly dispersed in the GelMA aqueous solution for polymerization. To analyze the mechanical properties of GelMA hydrogels (6%, w/v) with or without PEDOT NPs, the samples were crosslinked to analyze their complex storage modulus, respectively (Supplementary Fig. S2). Interestingly, the complex storage modulus decreased with the increase of the PEDOT NPs concentration ranging from 0.5 to 2.0 mg/ml. The deepened color of the composite hydrogel with the increased addition of PEDOT NPs made the samples absorbed the UV light, leading to incomplete crosslinking, as well as the decrease of the storage modulus. Subsequently, the swelling performance of the GelMA/PEDOT composite hydrogel was measured. As shown in Fig. 3B, the swelling ratio of the hydrogel decreased with the increase of PEDOT NPs, attributing to the hydrophobic property of PEDOT. Consequently, the composite hydrogel with PEDOT NPs showed reduced water-absorption performance compared with pure GelMA. However, the swelling ratio of composite hydrogel showed a barely decreasing trend while the concentration of PEDOT NPs higher than 1.5 mg/ml. The excess concentration of PEDOT NPs may absorb the light and disturb the UV irradiation, thus leading to the incomplete photo-crosslinking of the hydrogel. Consequently, the swelling ratio showed no significant difference while the concentration of PEDOT NPs higher than 1.5 mg/ml. Furthermore, to investigate the distribution of PEDOT NPs in GelMA, the freeze-dried GelMA/PEDOT composite hydrogels were observed by scanning electron microscope (SEM, S-4800, Hitachi, Tokyo, Japan) (Fig. 3C). Although the porous structure of GelMA/PEDOT composite hydrogel was uneven compared with pure GelMA, the surface of the composite was relatively slackened. Moreover, the surface of pure GelMA was smooth even under 80 000 times magnification. Contrarily, the surface of GelMA/PEDOT composite hydrogel became rough due to the presence of PEDOT NPs. In addition, the composite scaffold seemed to be denser with the increase of the PEDOT concentration.

Further, the rheological properties of GelMA hydrogels and GelMA/PEDOT NPs composite hydrogels were evaluated in terms of optimal concentrations of GelMA and PEDOT NPs (Supplementary Fig. S3). Since the velocities of the hydrogel solutions play significant roles in the printability and extrusion of inks, we evaluated the viscosity as a function of temperatures using GelMA aqueous solution without PEDOT NPs (Fig. S3A) [50]. It was evident that the viscosity values were increased with an increase in the GelMA concentration at the fixed temperature. In addition, the viscosity values of GelMA precursors at the various concentrations (4, 6, 8, and 10%, w/v) showed decreasing trends with the increase of temperature ranging from 17 to 40°C, while the high concentration of GelMA at 10% (w/v) showed more obvious thermos-responsive behavior than the GelMA precursors at 4, 6 and 8% (w/v). Considering the feasibility of printing, 6% (w/v) GelMA was determined, ensuring stable extrusion and shaping. Furthermore, the temperature dependence of the composite hydrogels was measured at 6% (w/v) GelMA at various concentrations of PEDOT NPs (Supplementary Fig. S3B). The viscosity of 6% GelMA (w/v) inks slightly increased with the increase of PEDOT NPs concentration. Moreover, the *G*′ and *G*″ values of the solutions with the variation of the temperature indicated a gelation temperature of 6% GelMA alone was approximately the same as the GelMA with the presence of PEDOT NPs at various concentrations (0.5, 1.0, 1.5 and 2.0 mg/ml), revealing the significant role of GelMA for gelation (Supplementary Fig. S3C) [50]. Further, the shear-rate dependence behavior of GelMA (6%, w/v) with PEDOT NPs (1.0 mg/ml) at 15 and 25°C was demonstrated, ensuring the feasibility of extrusion of composite hydrogel (Supplementary Fig. S3D). Together, considering the sufficient conductivity and photo-crosslinking of the composite hydrogel, the GelMA at 6% (w/v) and PEDOT NPs at 1.0 mg/ml were determined optimal for printing.

**Figure 4.**
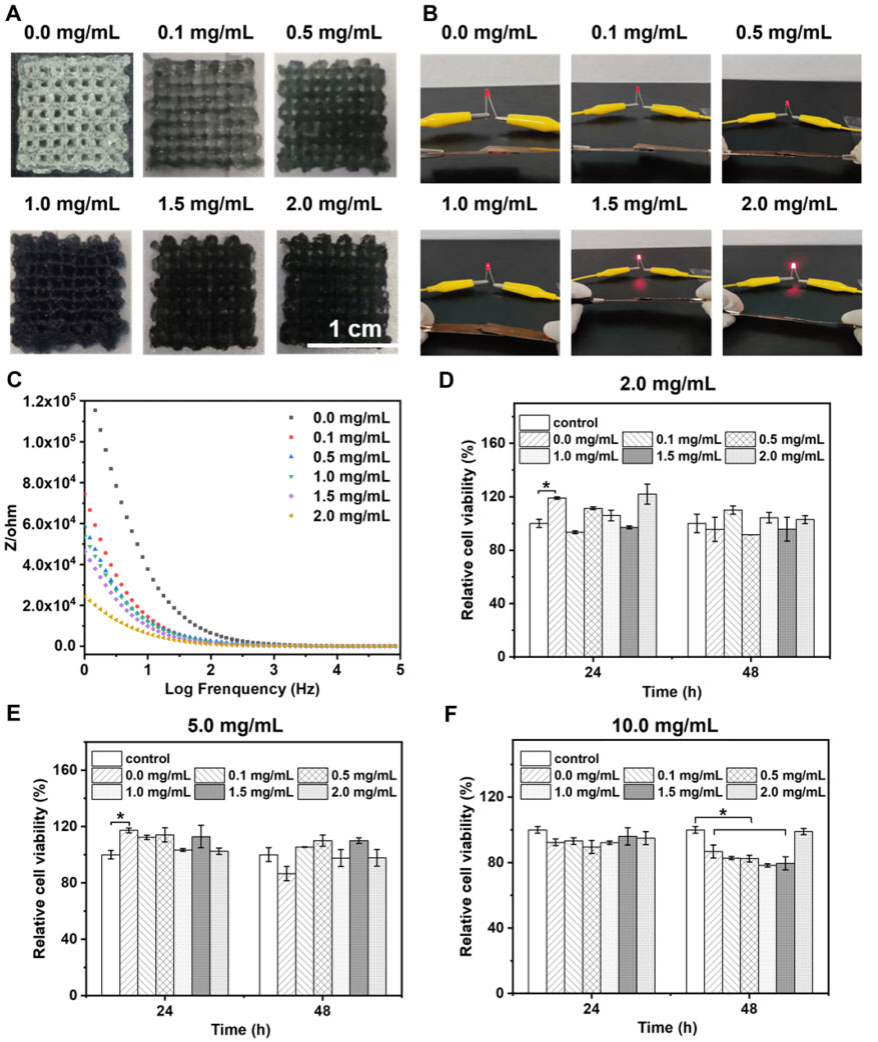
Characterization of 3D printing GelMA/PEDOT hydrogel scaffolds. (**A**) Photographs of GelMA/PEDOT hydrogel scaffolds, (**B**) light-emitting diodes forming a closed loop and (**C**) impedance diagram of GelMA/PEDOT hydrogel scaffolds printed at different concentrations of PEDOT NPs (0.0, 0.1, 0.5, 1.0, 1.5, and 2.0 mg/ml). (**D**–**F**) Cytotoxicity tests *in vitro* demonstrating the viabilities of C2C12 cells after incubation with (D) 2, (E) 5 and (F) 10 mg/ml of GelMA/PEDOT leach solution at 24 and 48 h, respectively (*P < 0.05).

The GelMA/PEDOT composite scaffold could be successfully printed using 3D bioprinting technology. In addition, the scaffold without PEDOT NPs was transparent with a smooth surface (Fig. 4A). However, the color of the scaffolds became darker with the increase of PEDOT concentration and eventually turned black with a rough surface. Together, the color change of scaffolds after printing presented the same trend as hydrogel discs. Notably, the space of the scaffold was arranged in an orderly manner, and there was precisely room in the space, which could be essential for the transportation of nutrients in the medium.

Subsequently, we examined the conductivity of the GelMA with or without PEDOT NPs. A hydrogel disc (diameter: 15 mm, thickness: 2 mm) was placed between two copper sheets; thus, a closed loop was formed with a wire and a light-emitting diode to provide a direct current voltage of 5 V [51, 52]. As depicted in Fig. 4B, the diode connected with pure GelMA hydrogel was extinguished, indicating a merely conductive property of pure GelMA. Furthermore, we measured the conductivity of GelMA hydrogel with various concentrations of PEDOT NPs (0.5, 1.0, 1.5, and 2.0 mg/ml) (Supplementary Table S1). It was observed that the conductivity of GelMA/PEDOT composite hydrogel increased with the increase of the concentration of PEDOT NPs. Notably, the conductivity of the composite hydrogel was close to that of normal tissue (from 5 × 10^−5^–1.6 × 10^−3^ S/cm) while the concentration of PEDOT NPs at 1.0 mg/ml [53]. Moreover, the altering current (AC) impedance of GelMA/PEDOT hydrogels was analyzed to investigate the capacitive and resistive electron transfers at the interface of biological materials [51]. Subsequently, the AC impedance of GelMA/PEDOT hydrogels was analyzed to investigate the capacitive and resistive electron transfers at the interface of biological materials. It was observed that capacitive currents dominated at higher frequencies, and all GelMA/PEDOT hydrogels exhibited low impedance (Fig. 4C). However, the resistive current dominated at lower frequencies was similar to a biological tissue at 1 Hz. GelMA/PEDOT hydrogels had presented lower impedance than pure GelMA hydrogels, indicating that the addition of PEDOT NPs could enhance the hydrogel’s conductivity.

Prior to demonstrating the performance efficacy *in vitro*, we investigated the cytotoxicity of the leaching solution of GelMA/PEDOT hydrogel by CCK-8 assay (Fig. 4D–F). Compared to the blank control group using a complete fresh medium, the survival rate of cells in the treatment groups (scaffold extract concentration of 2, 5, and 10 mg/ml along with PEDOT in the scaffold, 2 mg/ml) was greater than 75%, indicating no severe cytotoxicity to C2C12 cells. Consequently, these results showed that GelMA/PEDOT composite scaffold could be suitable for subsequent cell experiments *in vitro*.

### 3D Bioprinting of GelMA/PEDOT composite scaffold

**Figure 5.**
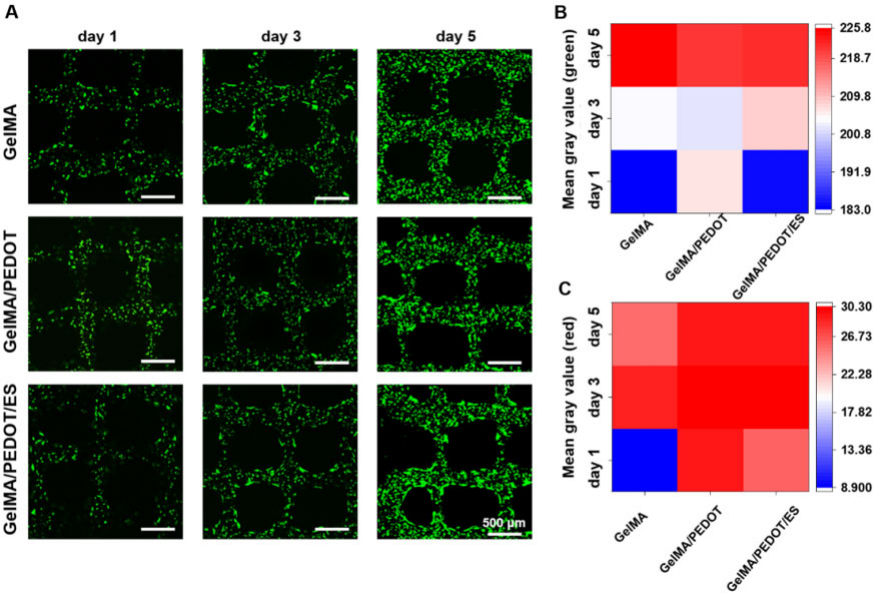
The viabilities of the encapsulated C2C12 cells within GelMA, GelMA/PEDOT NPs and GelMA/PEDOT NPs composite hydrogel under ES. (**A**) Live (green)/dead (red) staining of C2C12 cells in the GelMA/PEDOT hydrogel scaffolds at various culture times (1, 3 and 5 days). (**B**) Mean gray value of live (green) cells and (**C**) dead (red) cells within GelMA, GelMA/PEDOT NPs and GelMA/PEDOT NPs-based composite hydrogels under ES at various culture times (1, 3 and 5 days).

C2C12 cells were encapsulated in GelMA scaffolds with or without PEDOT for 1, 3 and 5 days, respectively. Subsequently, the ES was applied to the scaffolds, and the live/dead cells were stained (Fig. 5). The apparent green fluorescence revealed the high cell viabilities of C2C12 cells on GelMA and GelMA/PEDOT scaffolds, attributing to the convenient transportation of nutrients within the line-shaped network structure of the scaffolds. In addition, few dead cells (red fluorescence) in the scaffold were observed, and the mean gray values of red fluorescence were relatively low, indicating the biocompatibility of the GelMA hydrogel and PEDOT NPs at 1.0 mg/ml. Notably, the cells became fusiform in the edge of the scaffold with the ES group from the live/dead staining on day 5, which demonstrated that a certain degree of ES could promote the expansion, proliferation and migration of C2C12 cells in the scaffolds. It was observed from the experimental results that the C2C12 cells showed a high survival rate on GelMA and GelMA/PEDOT scaffolds (green fluorescence), with a few dead cells (red fluorescence) in the scaffold, attributing to the line-shaped network structure of the scaffolds convenient for nutrients transportation. Moreover, it should be noted that the cells became fusiform in the edge of the scaffold with the ES group from the live/dead staining on day 5, indicating that a certain degree of ES could promote the expansion, proliferation and migration of cells in the scaffolds.

**Figure 6.**
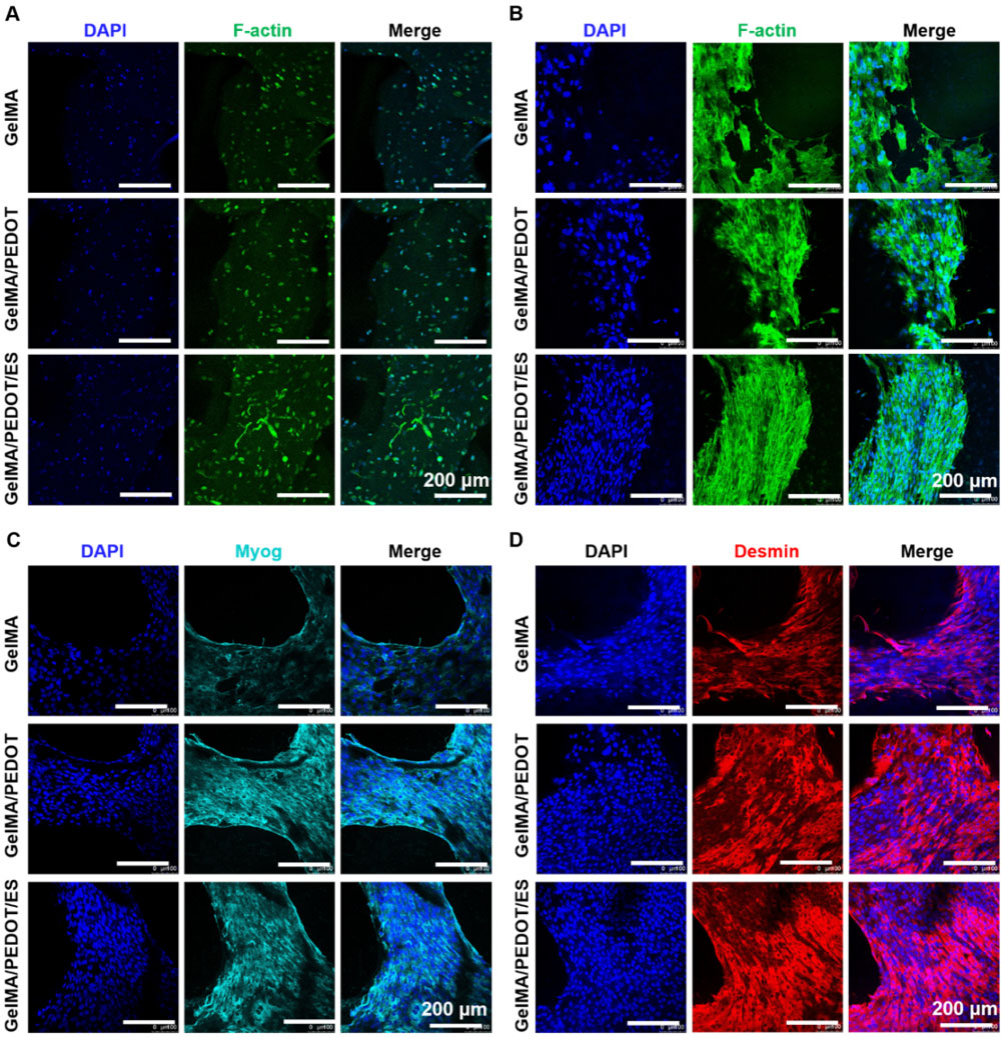
F-actin immunofluorescence staining of C2C12 cells within GelMA/PEDOT hydrogel scaffolds after (**A**) 3 days and (**B**) 10 days of culture. (**C**) Myog and (**D**) desmin immunofluorescence staining of C2C12 cells after 10 days of culture.

The formation of multinucleated myotubes involved initial adherence of mononuclear myoblasts and subsequent membrane arrangement. Consequently, the proliferation and migration of myoblasts determine the speed of myoblasts fusion [54]. However, the fusion of myoblasts was sometimes limited under natural physiological conditions. In this case, the ES could change the bioelectric field between cells and tissues, promoting cell proliferation and differentiation and ultimately inducing cell fusion into multinucleated myotubes. The F-actin staining indicated no apparent migration and fusion of C2C12 cells at 3 days of culture within GelMA scaffolds with or without PEDOT NPs (Fig. 6A). However, the C2C12 cells were migrated to the periphery of the scaffolds from DAPI fluorescent staining (blue) of the nucleus cultured for 10 days (Fig. 6B). In addition, the C2C12 cells stretched out, and the cytoskeleton became more significant from F-actin staining (green). Specifically, the C2C12 cells showed a more apparent orderly arrangement of the nucleus and cell fusion after ES treatment, indicating the enhanced formation of myotube at 10 days of culture. Consequently, these highly conductive GelMA/PEDOT-based scaffolds were more suitable for the proliferation and fusion of C2C12 cells than the pure GelMA scaffold.

Desmin is one of the earliest structural proteins detected during myogenesis. In mature muscle fibers, desmin is located around the nuclear membrane and the sarcolemma as a marker of myogenesis [55]. Myog expresses at the end of the myogenic differentiation and plays a crucial role in regulating the myogenic process and forming muscle fibers [55]. Myog and desmin immunofluorescence staining of the C2C12 cells were performed after culturing within the pure GelMA, GelMA/PEDOT NPs and GelMA/PEDOT NPs with ES treatment for 10 days, respectively. Myog and desmin immunofluorescence staining of the C2C12 cells were performed after culturing within the scaffold for 10 days. It was observed that the expressions of Myog (Fig. 6C) and desmin (Fig. 6D) were positive, indicating the maturation and differentiation of the C2C12 cells within the hydrogel scaffold after 10 days of culture. Moreover, the fluorescence intensities of Myog and desmin with the presence of PEDOT NPs were much higher than that of pure GelMA scaffold, demonstrating the essential role of conductive materials within hydrogel scaffolds.

## Conclusion

In summary, we prepared a conductive hydrogel scaffold based on GelMA and PEDOT NPs, to improve the proliferation of C2C12 cells and the formation of muscle fibers. The hybrid bioink containing GelMA and PEDOT NPs could be printed using 3 D extrusion bioprinting with excellent structure stability, high water absorption, favorable biocompatibility and good resolution. In addition, the C2C12 cells encapsulated in the fabricated GelMA/PEDOT conductive scaffold could better proliferate, migrate and differentiate compared to those in the pure GelMA scaffold. Furthermore, ES could also promote the myogenic differentiation of C2C12 cells in the scaffolds with PEDOT NPs. Together, we believe that the fabrication of the conductive hydrogels provides a feasible approach for the encapsulation of cells and the regeneration of the muscle tissue.

## Supplementary data

Supplementary data are available at *REGBIO* online.

## Funding

This study received financial support from the National Natural Science Foundation of China (NSFC, 32071323, 81971734 and 31800794), National Key R&D Program of China (2018YFB1105600), Natural Science Foundation of Fujian Province (2019J01076), the support by the Fundamental Research Funds for the Central Universities (ZQN-713), Funds for Foreign Experts from Ministry of Science and Technology, China (G20190013023) and Program for Innovative Research Team in Science and Technology in Fujian Province.

*Conflict of interest statement*. None declared.

## Supplementary Material

rbab035_Supplementary_Data
