# Supplementary material for: 3D bioprinting of conductive hydrogel for enhanced myogenic differentiation
Source: Regen Biomater. 2021 Aug 14;8(5):rbab035. doi: 10.1093/rb/rbab035 (PMC8363764; doi:10.1093/rb/rbab035)
Supplement: rbab035_Supplementary_Data [file rbab035_supplementary_data.docx]

*Supporting information*

**3D Bioprinting of Conductive Hydrogel for Enhanced Myogenic Differentiation**

**Ying Wang^1,2,†^, Qingshuai Wang^1,†^, Shengchang Luo^1^, Zhoujiang Chen^1,3^, Xiang Zheng^1,3^, Ranjith Kumar Kankala^1,3^, Aizheng Chen^1,3,*^, Shibin Wang^1,3,*^**

^1^ Institute of Biomaterials and Tissue Engineering, Huaqiao University, Xiamen 361021, P. R. China

^2^ School of Pharmaceutical Engineering and Life Science, Changzhou University, Changzhou 213164, P. R. China

^3^ Fujian Provincial Key Laboratory of Biochemical Technology (Huaqiao University), Xiamen 361021, P. R. China

* Correspondence address. Institute of Biomaterials and Tissue Engineering, Huaqiao University, Xiamen 361021, P. R. China. Emails: [azchen@hqu.edu.cn](mailto:azchen@hqu.edu.cn) (A. C.); sbwang@hqu.edu.cn (S. W.)

^†^ These authors contributed equally to this work.


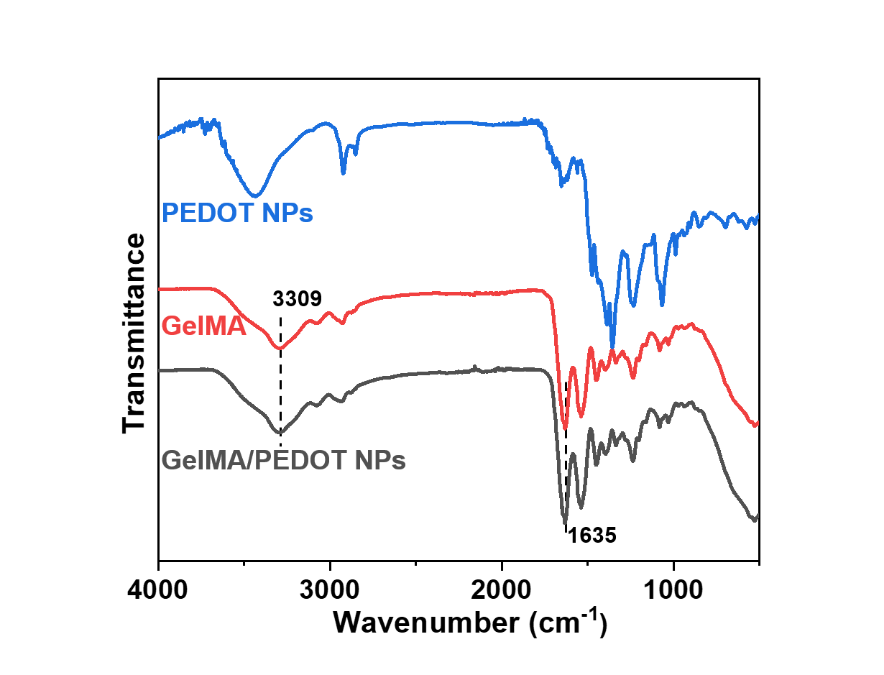


Figure S1. FTIR spectrum of PEDOT NPs, GelMA, and GelMA/PEDOT NPs composite hydrogel.


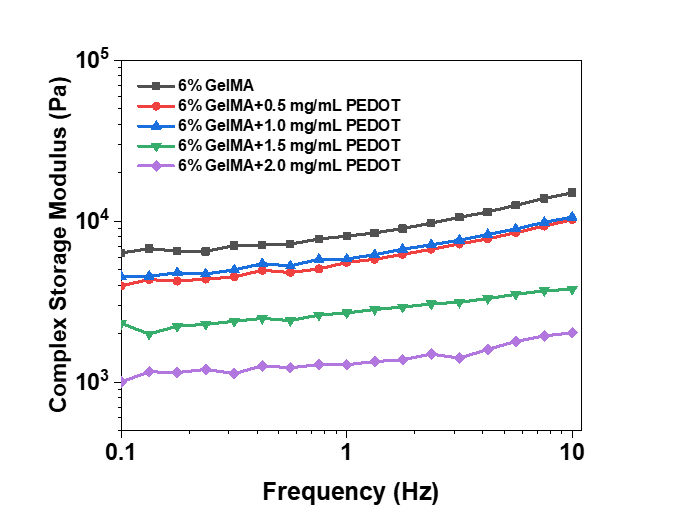


Figure S2. Mechanical properties of the GelMA hydrogel and GelMA/PEDOT NPs composite hydrogels.


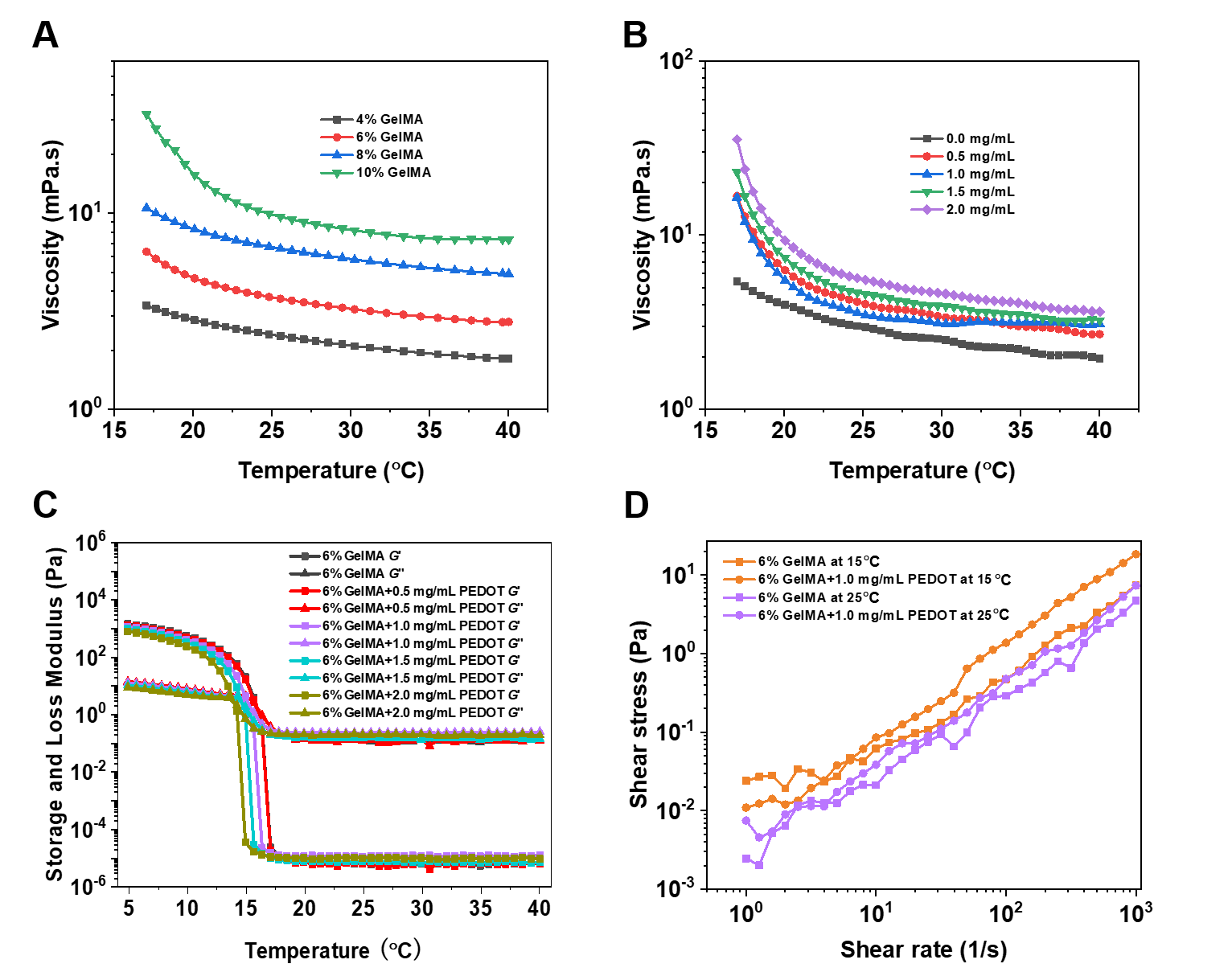


Figure S3. Rheological properties of the bioinks. (A) Viscosity as a function of temperature ranging from 17 to 40 ℃ at various GelMA concentrations (4, 6, 8, and 10%, w/v). (B) Viscosity as a function of temperature ranging from 17 to 40 ℃ at 6% (w/v) GelMA with various concentrations of PEDOT NPs (0.0, 0.5, 1.0, 1.5, and 2.0 mg/mL), respectively. (C) Effect of temperature on G’ and G” at 6% (w/v) GelMA with various concentrations of PEDOT NPs (0.0, 0.5, 1.0, 1.5, and 2.0 mg/mL), respectively. (D) Shear stress as a function of shear rate at 15 and 25 ℃, respectively, using GelMA (6%, w/v) with the addition of PEDOT NPs (1.0 mg/mL).

Table S1. Conductivity of GelMA/PEDOT NPs hydrogels

| Concentration (mg/mL) | 0.5 | 1.0 | 1.5 | 2.0 |
| --- | --- | --- | --- | --- |
| Conductivity (S/cm) | 5.21 × 10^-5^ | 9.75 × 10^-5^ | 2.12 × 10^-4^ | 1.19 × 10^-3^ |
